# Inflammation-Associated Synaptic Alterations as Shared Threads in Depression and Multiple Sclerosis

**DOI:** 10.3389/fncel.2020.00169

**Published:** 2020-06-23

**Authors:** Antonio Bruno, Ettore Dolcetti, Francesca Romana Rizzo, Diego Fresegna, Alessandra Musella, Antonietta Gentile, Francesca De Vito, Silvia Caioli, Livia Guadalupi, Silvia Bullitta, Valentina Vanni, Sara Balletta, Krizia Sanna, Fabio Buttari, Mario Stampanoni Bassi, Diego Centonze, Georgia Mandolesi

**Affiliations:** ^1^Synaptic Immunopathology Lab, Department of Systems Medicine, Tor Vergata University of Rome, Rome, Italy; ^2^Synaptic Immunopathology Lab, IRCCS San Raffaele Pisana, Rome, Italy; ^3^Department of Human Sciences and Quality of Life Promotion, University of Rome San Raffaele, Rome, Italy; ^4^Unit of Neurology, Mediterranean Neurological Institute IRCCS Neuromed, Pozzilli, Italy

**Keywords:** multiple sclerosis, major depressive disorder, excitotoxicity, antidepressant drugs, cytokines, synaptopathy, neuroinflammation, monoamine

## Abstract

In the past years, several theories have been advanced to explain the pathogenesis of Major Depressive Disorder (MDD), a neuropsychiatric disease that causes disability in general population. Several theories have been proposed to define the MDD pathophysiology such as the classic “monoamine-theory” or the “glutamate hypothesis.” All these theories have been recently integrated by evidence highlighting inflammation as a pivotal player in developing depressive symptoms. Proinflammatory cytokines have been indeed claimed to contribute to stress-induced mood disturbances and to major depression, indicating a widespread role of classical mediators of inflammation in emotional control. Moreover, during systemic inflammatory diseases, peripherally released cytokines circulate in the blood, reach the brain and cause anxiety, anhedonia, social withdrawal, fatigue, and sleep disturbances. Accordingly, chronic inflammatory disorders, such as the inflammatory autoimmune disease multiple sclerosis (MS), have been associated to higher risk of MDD, in comparison with overall population. Importantly, in both MS patients and in its experimental mouse model, Experimental Autoimmune Encephalomyelitis (EAE), the notion that depressive symptoms are reactive epiphenomenon to the MS pathology has been recently challenged by the evidence of their early manifestation, even before the onset of the disease. Furthermore, in association to such mood disturbance, inflammatory-dependent synaptic dysfunctions in several areas of MS/EAE brain have been observed independently of brain lesions and demyelination. This evidence suggests that a fine interplay between the immune and nervous systems can have a huge impact on several neurological functions, including depressive symptoms, in different pathological conditions. The aim of the present review is to shed light on common traits between MDD and MS, by looking at inflammatory-dependent synaptic alterations associated with depression in both diseases.

## Introduction

Depression is one of the most diagnosed mental disorders in adult patients as well as in children and adolescents. It is a heterogeneous disorder caused by a complex interaction between genetic and environmental factors (Wang et al., 2015; Fakhoury, 2016; Otte et al., 2016; Shadrina et al., 2018). Stress is a major risk factor for depression, in particular early life stress, and enduring stress exerted on susceptible individuals may ultimately lead to Major Depressive Disorder (MDD) and eventually suicide behavior (Bielau et al., 2005; Otte et al., 2016; Tauil et al., 2018). Pathophysiological hallmarks of depressive symptoms include monoamine depletion, glucocorticoid receptor (GR) resistance, corticotrophin- releasing hormone and cortisol levels as well as excess of glutamate (Boku et al., 2018; Seki et al., 2018). Several theories, such as the classic ‘monoamine-theory’ as well as the ‘glutamate hypothesis’ have been proposed to define the molecular and cellular mechanisms underlying this pathological condition (Paul-Savoie et al., 2011; Albert et al., 2012). Over the last years, all these theories have been challenged by evidence highlighting inflammation as a pivotal player in developing depressive symptoms (Alesci et al., 2005). In particular, proinflammatory cytokines have been claimed to play a role also in stress-induced mood disturbances and in major depression, indicating a widespread role of classical mediators of inflammation in emotional control (Khandaker et al., 2018). Moreover, during systemic inflammatory diseases, peripherally released cytokines circulate in the blood, reach the brain and cause anxiety, anhedonia, social withdrawal, fatigue, and sleep disturbances (Raison et al., 2006; Dantzer et al., 2008; Miller et al., 2009). Accordingly, chronic inflammatory diseases, as cardiovascular disease, type 2 diabetes, cancer, psoriasis, rheumatoid arthritis and the inflammatory autoimmune disease multiple sclerosis (MS), have been associated to high incidence of depressive symptoms and higher risk of MDD (Poole and Steptoe, 2018) in comparison with overall population (Raison et al., 2006; Finnell and Wood, 2016; Poole and Steptoe, 2018).

How inflammation contributes to depressive symptoms either in chronic inflammatory disease or in the context of MDD has been highly debated (Haroon et al., 2012) and is still under intense investigation considering the potential impact on therapy decision making and improvement of patients quality of life (Otte et al., 2016). Notably, in the inflammatory autoimmune disease MS and in its mouse model, namely experimental autoimmune encephalitis (EAE), it is emerging that proinflammatory cytokines may directly alter neuronal activity and cause mood alterations independently of their destructive effects on myelinated axons and of the associated disability. An early and diffuse inflammation-dependent enhancement of glutamatergic transmission as well as synaptic plasticity perturbation characterize the EAE/MS brains, resembling some of the MDD pathological hallmarks.

**FIGURE 1:**
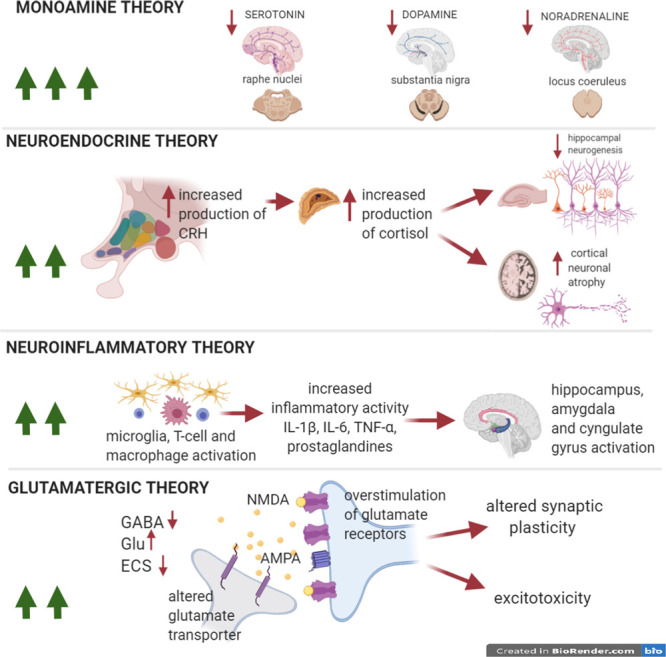
Pathophysiological theories in MDD. The numebr of green arrows is proportional to the impact of each theory on the pathophysiology of the disease. Pathophysiological theories to explain the onset of depressive symptoms in MDD. The so-called monoamine hypothesis explains depression as a result of selective monoamine depletion (serotonin, dopamine, and noradrenaline) in several areas of the CNS. The neuroendocrine and neuroinflammatory theories could equally well explain depressive symptoms in MDD. The first hypothesis implies the hyperactivity of the hypothalamic-pituitary-adrenal axis (HPA), with an increase in the production of cortisol and a consequent reduction in hippocampal neurogenesis and plasticity in cortex. The second hypothesis emphasizes microglial activation mechanisms and the resulting neuroinflammatory response, that causes, in turn, hyperactivation of eloquent areas of the limbic system (amygdala, hippocampus, and cingulate gyrus). In addition, the glutamatergic theory postulates that also this neurotransmitter is relevant for the formation of depressive symptoms and cognitive impairment, through the overstimulation of glutamate NMDA receptors, the alteration of AMPA phosphorylation, impaired glutamate reuptake, and ECS dysfunction. The long-lasting effects will be excitotoxic damage, and synaptic plasticity impairment. The neuroinflammatory and glutamate theories are strictly interconnected with the inflammatory-synaptopathy hypothesis, as suggested in MS studies.

**FIGURE 2:**
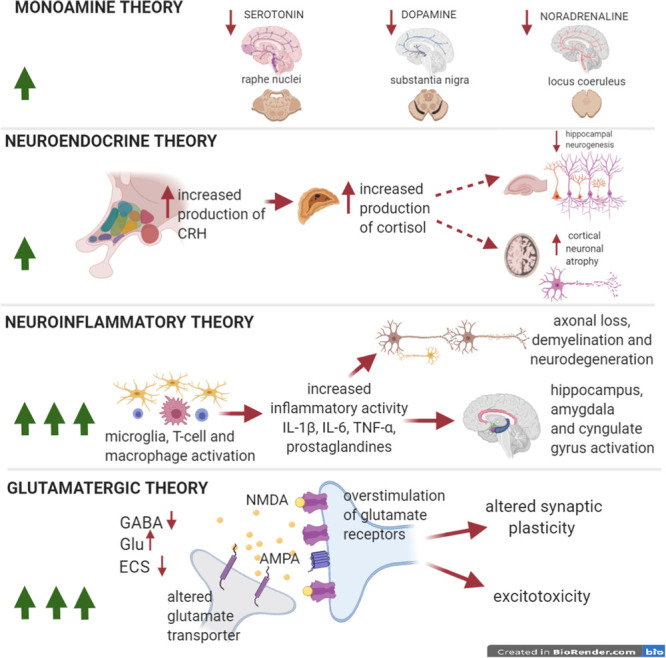
Pathophysiological theories of depression in MS. The number of green arrows in windows is directly proportional to the importance of each theory in the pathophysiology of the disease. Pathophysiological theories to explain the onset of depressive symptoms in MDD. Depression in the course of MS/EAE presents a wide range of clinical presentations. Compared to MDD, monoamine dysfunction is likely less relevant, although it is fundamental in the genesis of depressive symptoms, as it is supported by a good pharmacological response to SSRIs (anti-inflammatory effect). The neuroendocrine theory, responsible of HPA-cortisol perturbation is less investigated in MS than in MDD. The neuroinflammatory theory stands out of importance in MS depressive disorder, sustained by activated T cells and macrophage/microglia that secrete proinflammatory cytokines, causing demyelination, axonal loss a neurodegeneration. Perturbation of limbic areas has been associated to microgliosis. Finally, the glutamate theory is essential in understanding depressive symptoms associated to MS/EAE, especially in the early phase of the disease even before clinical onset. Increased hyperexcitability, caused by enhanced glutamatergic transmission and by a reduced GABAergic tone, has been associated to early mood disturbances. Altered synaptic plasticity also causes cognitive impairment. The link between the neuroinflammatory theory and the glutamatergic hypothesis has been well characterized in MS and the inflammatory synaptopathy has been recognized as a reliable hallmark of MS/EAE.

**FIGURE 3:**
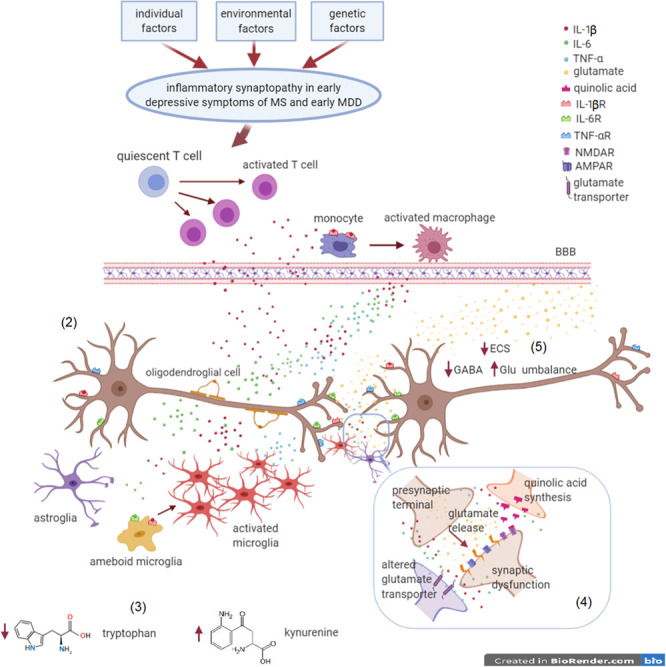
Inflammatory synaptopathy in early depressive symptoms of MS and MDD. MS and MDD are different brain diseases, induced by (1) environmental factors, genetic susceptibility and individual factors (most of these factors are still unknown and apparently not in common). In the early MS and MDD courses, a chronic inflammatory state can promote synaptic dysfunctions in brain areas involved in mood control, leading to depressive symptoms. (2) Immune dysfunction driven by T cells and monocytes/macrophages activation as well as by microgliosis in the CNS is responsible of detrimental synaptic dysfunctions. Proinflammatory cytokines, such IL-1β, TNF-α, and IL-6, are the main players of the crosstalk between the immune and the nervous system. (3) Glial cells lead to tryptophan conversion into kynurenine, a metabolite at the basis of the synthesis of quinolic acid. (4) Quinolic acid, produced by activated microglia, stimulates post-synaptic NMDA glutamate receptors, with an excitotoxic effect. (5) Moreover, proinflammatory cytokines cause an overstimulation of AMPA receptors and an impairment of glutamate reuptake, responsible of enhanced glutamatergic transmission. Hyperexcitability is also a consequence of a low GABAergic tone and ECS impairment. These alterations exacerbate excitotoxicity and lead to synaptic plasticity perturbation, eventually resulting in neurodegeneration.

Although analogies between a neuropsychiatric disorder such as MDD and the inflammatory autoimmune MS disease are difficult to sustain, neuroinflammation is potentially recognized as a common disease factor. In this regard, it is worth considering the huge effect that small physiologic differences of inflammatory components can have over time if they are consistently skewed in one direction. The aim of the present review is to provide an overview of the several theories advanced to explain depressive symptoms in MDD and in MS (Figures 1, 2), with a particular emphasis on the role of inflammation and on its impact on synaptic alterations in mood disturbances (Figure 3). Ultimately, insights on clinical implications will be provided in the context of the cytokine and glutamate theories.

## Pathophysiology of MDD

Major depressive disorder is one of the most common psychiatric disorder in the world recognized by the World Health Organization (WHO) and affects general population across a broad spectrum of ages and social environmental factors (Malhi and Mann, 2018).

The one-year prevalence of MDD is 6% representing about twofold greater incidence in women than man (Malhi and Mann, 2018). The lifetime risk of developing MDD for a person is 15–18% and almost 40% of MDD population experiences the first episode before 20 years (Belmaker and Agam, 2008). MDD is mainly characterized by depressed mood and anhedonia as fundamental symptoms but it may be associated to several other clinical features including anorexia, weight loss, insomnia, hypersomnia, and suicidal thoughts. Moreover, other affections, including cognition, memory and motor dysfunctions have been associated to MDD (Malhi and Mann, 2018).

Clinical features coincide with the evidence of morphological and functional alterations of gray and white matter in the brain of MDD patients. In the recent years, magnetic resonance imaging (MRI) studies revealed cortical thinning in several brain regions of MDD patients including frontal lobe, temporal lobe, hippocampus, pre-frontal cortex (PFC), anterior cingulate cortex (ACC), orbito-frontal cortex, thalamus, and striatum (Suh et al., 2019). Worthy of note, alterations in the hippocampus, a brain area associated to cognition and memory, are hallmarks of MDD. This has also been supported by a large scale meta-analysis of structural neuroimaging studies showing that hippocampal abnormalities were more frequently discovered in MDD patients when compared to other psychiatric disorders (e.g., schizophrenia and bipolar disorder) and to healthy controls (Goodkind et al., 2015). Among neuroimaging investigations, functional MRI (fMRI) studies explore functional activity and connectivity between specific brain regions of interest measuring the extent of synchronization in the spontaneous fluctuations of blood oxygenation level dependent (BOLD) signal. In MDD patients, altered connectivity is frequently reported in several brain regions, including amygdala, frontal cortex, ACC and ventral striatum by BOLD-fMRI studies (Otte et al., 2016). Those studies suggest that MDD pathophysiology is not only associated to macroscopic alterations of specific brain areas but is also due to their dysfunctional activity (Figure 1). In the next chapters we will discuss the neurobiological bases of these phenomena.

## MDD Pathophysiology Hypothesis: the Monoamine Theory and the Neuroendocrine Hypothesis

Various hypotheses have been proposed to explain the pathophysiology of MDD (Boku et al., 2018) (Figure 1). The most common is the monoamine hypothesis, based on the evidence that the concentrations of monoamines, such as serotonin, noradrenaline and dopamine, in synaptic gaps are decreased in the depressive state (Boku et al., 2018). The neurotransmitter 5-hydroxytryptamine (5-HT), namely serotonin, is implicated in the regulation of mood and pain. This monoamine is diffusely located in the whole body especially in the enterochromaffin cells of the gastrointestinal tract, in the platelets and in the central nervous system (CNS). It is synthetized from the essential amino acid L-tryptophan by the enzyme tryptophan hydroxylase in neurons located in the brainstem raphe nuclei (Mohammad-Zadeh et al., 2008). Conversely, the dopamine (DA) and noradrenaline (NA) neurotransmitters belong to the group of catecholamines and are derived from the amino acid L-tyrosine. Dopaminergic neurons originate from both the substantia nigra pars compacta and ventral tegmental area (VTA) and project to the dorsal and ventral striatum, respectively. Dopaminergic system is implicated in the motivation, addiction and motor function (Klein et al., 2019). Finally, NA released by noradrenergic neurons located in the locus coeruleus (LC) is implicated in modulation of arousal state and represents an important part of the stress response (Schwarz and Luo, 2015).

The modulation of monoamine synaptic transmission is still the main target of pharmacological treatment in MDD (Fakhoury, 2016). As historically demonstrated in the 1960s, depressive symptoms can be controlled by inhibiting several molecules involved into monoamine distribution and metabolism in the synaptic compartment. Indeed, the main target of antidepressants includes the serotonin and noradrenaline transporters (SERT and NET) involved in the re-uptake of monoamines, commonly blocked by tricyclic antidepressant (TCA) and more specifically by selective serotonin reuptake inhibitors (SSRI), or the metabolizing enzyme of monoamine, namely mono-amine oxidase (MAO), blocked by the MAO inhibitors (iMAO). The main neurotransmitter involved into MDD pathophysiology is serotonin, as demonstrated by clinical studies showing reduced serotonin metabolites in biological fluids and genetic variants associated with SERT in MDD patients (Dean and Keshavan, 2017). The role of NA in MDD is still controversial, while increasing NA levels by transporter inhibition with NA-reuptake inhibitors (NARI) ameliorates depressive symptoms, conversely, elevated NA levels in the CNS have been associated to stress and depressive symptoms (Seki et al., 2018). In the last years, the validity of the monoaminergic theory has been largely debated. Of note, it has been observed that antidepressants are rapidly effective on monoamine synaptic concentration, but the onset of therapeutic effect generally takes 2–4 weeks (Boku et al., 2018). Moreover, experimental depletion of NA and serotonin did not induce depressive symptoms in healthy individuals but only in previously depressed patients treated successfully with antidepressant drugs (Ruhé et al., 2007). Furthermore, 30% of MDD patients are resistant to antidepressant treatments (Boku et al., 2018). These findings strongly suggest that monoamine impairment is partially responsible of MDD development, and that additional neurobiological factors (i.e., second messengers and their cascade systems activation), environmental factors (i.e., socioeconomic status, early life stress, and violence exposure), chronic stress and genetic susceptibility (influencing early onset, recurrence, and severity of the disease) are required to fully trigger the pathological status (Wang et al., 2015; Fakhoury, 2016; Otte et al., 2016; Shadrina et al., 2018). Among these, chronic stress is the most important causal agent for MDD. Pre-clinical studies conducted on murine model also confirmed this causality. A chronic stress, such as exposure to a long-term inescapable stimulation or a chronic social defeat, is indeed able to induce depressive symptoms in rodents as revealed by specific behavioral tests such as forced swimming test, tail suspension test and sucrose preference test (Hao et al., 2019). Although modeling human depression in animals is challenging and present several limitations, rodent models have been useful in describing specific aspects of mood disturbances in well-designed experimental settings (Hao et al., 2019).

Early life stress is known to have an impact on neuroendocrine regulation and neurodevelopment; a neuroendocrine theory has been indeed proposed to explain depressive symptoms in MDD patients. Stress activates the hypothalamic-pituitary-adrenal axis (HPA) inducing hypothalamic release of corticotrophin releasing hormone (CRH). CRH stimulates anterior pituitary gland to release adrenocorticotropic hormone (ACTH) leading to glucocorticoid secretion by adrenal glands. This HPA hyperactivity has been associated with increased levels of glucocorticoids both in the plasma and in the cerebrospinal fluid (CSF). The hippocampus plays a key role in the feed-back regulation of HPA and increased CNS level of glucocorticoids overstimulates hippocampal neurons leading to volume decrement. Two main hypotheses have been proposed to explain hippocampal alterations: the neurogenesis hypothesis, in which glucocorticoids decrease the neurogenesis in the dentate gyrus (DG) of the hippocampus, and the neuroplasticity hypothesis, in which glucocorticoids induce the atrophy of mature neurons reducing the total dendritic spine density and length. These structural and functional alterations seem to be associated to lower levels of brain-derived neurotrophic factor (BDNF) in the hippocampus and have been proposed as the neural basis of cognitive alterations in chronic stress and MDD (Boku et al., 2018).

## The Neuroinflammatory Theory in MDD

Accumulating evidence has associated chronic inflammation to MDD and to chronic stress pathophysiology, but it is still a matter of debate how inflammation may contribute to the pathogenesis of these diseases (Kim and Won, 2017). As reported in the following paragraphs, alterations of immune cells, including lymphocytes, monocytes/macrophages and microglia, as well as soluble inflammatory mediators have been associated to several depressive states (Petralia et al., 2020) (Figures 1, 3).

### The Cytokine Theory of MDD

The involvement of cytokines in psychiatric diseases was first postulated through the observation that patients treated with the pro-inflammatory cytokine IFN-α developed several depressive symptoms that disappeared when treatment was interrupted (Smith, 1991; Maes, 1995).

In 1991, Smith proposed the so-called cytokine theory of MDD, supported by clinical and pre-clinical studies (Dey and Giblin, 2018). According to this theory, MDD patients exhibit a chronic inflammatory status characterized by elevated serum levels of pro-inflammatory cytokines (Figures 1, 3), including tumor necrosis factor α (TNF-α), interleukin (IL-) 1β, IL-2, IL-6, IL-12, IL-13 interferon gamma (IFN-γ), and decreased level of anti-inflammatory cytokines, such as IL-10 and soluble receptor for IL-2 and IL-6, IL-1β receptor antagonist (IL-1ra), in respect to healthy controls (Petralia et al., 2020).

Clinical studies have also suggested that serum levels of cytokines may provide several information both on the risk to develop the disease and on clinical outcome. For example, a retrospective study demonstrated that children with higher levels of IL-6 have 10% greater risk of developing MDD (Khandaker et al., 2014). Moreover, elevated levels of TNF-α and IL-6 are predictive of a higher risk of suicide and more severe course of the disease (Janelidze et al., 2011; Haapakoski et al., 2015). Similarly, MDD patients with elevated plasmatic levels of IL-6, IL-8, IFN-γ, and TNF-α manifest slight responsiveness to sleep deprivation, a commonly approved approach to treat depressive symptoms in MDD (Petralia et al., 2020). Of note, all these studies have been limited to few cytokines such as TNF-α, IL-1β, IL-6, and IFN-γ. Furthermore, several genetic polymorphisms of cytokines genes have been involved in MDD pathophysiology. It has been demonstrated that the single nucleotide polymorphism (SNP) rs1800629 of the TNF-α gene is associated to a higher risk of developing MDD especially in Alzheimer disease and post stroke patients (Zhou et al., 2018). An higher risk to develop MDD has been associated to the IL-6 receptor polymorphisms Asp358Ala and rs2228145 (Khandaker et al., 2014) and to the SNPs rs187238 and rs1946518 on the IL-18 promoter gene in patients that are exposed to stressful events (Haastrup et al., 2012; Petralia et al., 2020).

The association between mood disturbances and cytokine levels has been explored also in several mouse models. For example, a preclinical study on knock out (KO) mice for TNF-α receptor 1 or 2 (TNFR1-2 KO) showed amelioration of conditional fear response and depressive behavior in comparison to control mice (Ma et al., 2016) as well as intracerebral infusion of TNF-α in healthy mice induces mood disturbances (Haji et al., 2012). Moreover, intraperitoneal injection of lipopolysaccharide (LPS), a proinflammatory agent obtained from Gram-negative bacteria, induces in rodents a transient flu-like syndrome in association with the activation of the immune system and consequent release of inflammatory cytokines (including TNF-α, IL-1β, IL-6, and IFNγ) (Dantzer and Kelley, 2007; Brydon et al., 2008; Musaelyan et al., 2018). The induction of sickness behavior has been useful to investigate some MDD symptoms, such as fatigue, anorexia, anxiety, pain and sleep disorders, despite the limitation of being mainly an acute and transitory inflammatory response to an external agent. Accordingly, a state of chronic inflammation is detectable in murine models of depression induced by chronic stress exposure. Mice exposed to chronic social stress present increased levels of plasmatic IL-6 and increased brain levels of TNF-α, IL-1β, and IL-6 (Ménard et al., 2016; McKim et al., 2018; Deng et al., 2019). Furthermore, increased plasmatic levels of IL-6 can predict susceptibility to chronic social stress in adult mice (Ménard et al., 2016).

### The Role of Lymphocytes in MDD

It has been demonstrated that the main T helper phenotypes involved in the productions of pro-inflammatory cytokines in MDD are Th1, Th17, Th22 while reduced activity of Th2, Th3, Th9 has been reported (Petralia et al., 2020). These observations led some authors to propose an imbalance between Th1/Th17 and Th2/Th9 response at the basis of the MDD pathophysiology, reflecting an imbalance between pro-inflammatory and anti-inflammatory T lymphocyte phenotypes (Furtado and Katzman, 2015; Petralia et al., 2020). Unfortunately, this model is an oversimplification of a more complex mechanism. Recent studies suggest that differentiated T cells may turn into other T cell subsets, for example Th1 in specific conditions can release IL-10, while regulatory T cells can release IL-17 when expressing Foxp3 and RORγT, two genes commonly associated to Th17 (Petralia et al., 2020).

### The Role of Microglia in MDD

Microglia cells, resident immune cells of CNS, are potent and far-reaching regulators of the extended neuron-glia network by secreting soluble mediators and by establishing direct contacts with the synaptic compartment (Kettenmann et al., 2013; Bar and Barak, 2019). In neuroinflammatory conditions, monocytes/macrophages and activated microglial cells can have detrimental effects on synaptic structure and function and neuronal survival. In accordance to the MDD neuroinflammatory theory, these immune cells are largely involved in the pathophysiology of the disease and deeply influence its course (Dey and Giblin, 2018) (Figures 1, 3). Of note, a positron emission tomography (PET) study demonstrated increased distribution volume of the translocator protein (TSPO), suggesting a glial activation throughout several brain areas (cingulate gyrus, amygdala, prefrontal cortex, and hippocampus) deeply involved in MDD pathophysiology (Setiawan et al., 2015; Meyer, 2017). Accordingly, post-mortem studies found activated microglia in several brain areas including dorsolateral prefrontal cortex (DLPFC), hippocampus, ACC and amygdala. Moreover, in both studies microglial activity was associated to frequency of acute episodes, disease course, and suicide risk (Bielau et al., 2005; Khairova et al., 2009; Oh et al., 2012; Schnieder et al., 2014; Torres-Platas et al., 2014; Rubinow et al., 2016).

Many preclinical studies have demonstrated that activated microglia lead to an up-regulation of pro-inflammatory cytokines, such as IL-1β, TNF-α, and IL-6, prostaglandins, nitric oxide and glutamate causing functional and structural abnormalities of the synaptic compartment (Chen et al., 2018; Dey and Giblin, 2018). It has been demonstrated that, activated microglia may also indirectly contribute to cytokine release in brain tissue recruiting IL-1β releasing monocytes in mice exposed to chronic stress (McKim et al., 2018).

Interestingly, stress-induced activation of LC has been associated to increased NA levels in several projecting brain areas, including PFC and hippocampus (Seki et al., 2018), that in turn induce microglial activation mediated by α1- and β-adrenoceptors leading to the release of pro-inflammatory cytokines (Chen et al., 2018). Structural and functional alterations mediated by increased NA levels have been detected in association to cognitive impairment in chronic stress and MDD. In this regard, direct and indirect mechanisms have been proposed, NA neurotransmission potentiation induces not only microglia activation but exerts also an indirect effect by enhancing the release of CRH from the hypothalamus, influencing glucocorticoid levels and causing a synaptic dysregulation in the hippocampus of MDD patients (Pace et al., 2007; Pace and Miller, 2009; Wang et al., 2017). Moreover, elevated NA levels associated to fear response are reported in basolateral amygdala (BLA) and VTA, associated to anhedonia in MDD patients (Seki et al., 2018).

## Glutamatergic Theory, Structural and Functional Synaptic Alterations in MDD

Dysregulation between excitatory glutamatergic transmission and inhibitory GABAergic transmission is implicated in the pathophysiology of depression ascribing to the “glutamatergic theory of depression” that leads to excitotoxic damage, neurodegeneration and consequent brain damage (Lener et al., 2017; Meyer, 2017) (Figures 1, 3). Glutamate is the major excitatory neurotransmitter in the human brain and has a pivotal role in various brain functions and exerts its role by binding at glutamate receptors (GluRs). Neuronal GluR activation provides fast synaptic transmission activity and it is required for synaptic plasticity, learning and memory. The GluRs are represented by two main type of receptors, namely ionotropic glutamate receptors [*N*-methyl- D-aspartate (NMDA) receptors, α-amino-3-hydroxy-5-methyl-4-isoxazolepropionic acid receptors (AMPARs)], kainic acid receptors (KARs) and metabotropic glutamate receptors (mGluRs) expressed at both pre- and post-synaptic neurons (Popoli et al., 2012). In addition, the glial compartment of CNS, namely astrocytes, microglia, and oligodendrocytes exhibits GluR subtypes. To maintain homeostasis in the brain, the release of glutamate to the synaptic cleft is finely regulated by presynaptic metabotropic GluR (mGluR) activation or by inhibitory potential triggered by GABA but also from astrocytes through reuptake activity of excitatory amino acid transporters (EAATs).

Over the past decades, preclinical and clinical studies investigated the link between glutamatergic hyperactivity and the pathophysiology of depressive disorder, thus identifying the glutamate receptors (NMDA and AMPA) and glutamatergic synapse as a potential target for pharmacological treatments. Clinical studies suggest associations between glutamatergic hyperactivity and regional volume reductions in the brains of MDD patients as well as significant decrease in glial cell numbers, density and neuronal atrophy (Rajkowska et al., 1999; Sanacora et al., 2012). Furthermore, particularly relevant for the glutamate hypothesis of mood disorders is the association with specific SNPs in genes of GRIA3, GRIK4, GRIK2, and GRM7, which encode proteins of the AMPA, kainate and metabotropic receptors (de Sousa et al., 2017).

It is important to remind that the glutamate is an active player in synaptic brain plasticity (i.e., long term potentiation, LTP; long term depression, LTD) and cognitive impairment is considered as a core endophenotype of MDD (Hasler et al., 2004). Preclinical studies reported that exposure to stressful stimuli alters synaptic plasticity in animal models of MDD, suggesting that plasticity plays a key role in depressive behavioral state induction (Liu et al., 2015; Milior et al., 2016). NMDAR-dependent LTP is mediated by several signaling cascades activation including cAMP-dependent protein kinase (PKA), protein kinase C (PKC), the mitogen-activated protein kinase (MAPK) cascade that activates extracellular signal-regulated kinases (ERKs) and calcium/calmodulin-dependent protein kinase II (CaMKII) (Malenka and Bear, 2004). Genetic studies confirmed synaptic plasticity alterations in MDD by showing a constitutively decreased expression of components of the glutamatergic transmission machinery involved in glutamate homeostasis, such as glutamate transporters, and glutamine synthesis as well as oligodendrocyte and myelin related genes especially in frontal cortex tissue (Khairova et al., 2009; de Sousa et al., 2017).

The relevance of glutamatergic synaptic dysfunction in MDD is corroborated by pharmacological evidence that drugs with neuroprotective effects against excitotoxic mechanisms have antidepressant properties. Indeed, riluzole, by inactivating the voltage-dependent calcium and sodium channels exerts an inhibitory effect on glutamate release, has also antidepressant properties likely mediated by an enhancement of glutamate clearance by astrocytes, with consequent neuroprotective effects and synaptic plasticity preservation (Banasr and Duman, 2007). Similar results have been obtained in patients treated with ketamine, a high-affinity NMDA antagonist (Murrough et al., 2017). In a double-blind randomized clinical study, it has been demonstrated that patients suffering from treatment-resistant depression showed a rapid onset (i.e., after a single dose) antidepressant response to ketamine treatment with respect to untreated controls (Singh et al., 2016). Glutamatergic drugs have shed a light on the alternative possibility of treating MDD patients with a strong and rapid antidepressant effect induced by glutamatergic receptor antagonism. Accordingly, pre-clinical studies conducted on mice lacking various glutamatergic components (i.e., GLT-1 or GLAST proteins, NMDAR or AMPAR) as well as on behavioral mouse models, showed antidepressant effects mediated by modulation of synaptic glutamate concentrations (Li et al., 2011; Sanacora and Banasr, 2013; Fuchikami et al., 2015; Fullana et al., 2019a, b).

In addition, there is cumulating evidence that dysfunction of the GABAergic system is associated to the pathophysiology of MDD (Fogaça and Duman, 2019). By regulating the HPA axis, GABAergic neurons play an important role in the termination of stress response, and the failure of this regulatory path leads to anxiogenic and pro-depressive behaviors. Indeed, chronic stress exposure causes a disruption of GABA-mediated inhibition of the HPA axis by limiting the expression of the transmembrane K-Cl cotransporter (KCC2) (Hewitt et al., 2009). Moreover, it has been demonstrated that the downregulation of GABAA receptors in the frontal cortex and other limbic areas is involved in dendritic reorganization of interneurons (Gilabert-Juan et al., 2013) and alterations of electrophysiological responses (Northoff and Sibille, 2014). Of note, magnetic resonance spectroscopy (MRS) studies conducted on MDD patients reported reduced levels of GABA in several cortical areas and that a normalization of GABA levels is associated to the remission of depressive symptoms (Hasler et al., 2007; Godfrey et al., 2018).

Finally, components of the endocannabinoid system (ECS), such as the type-1 cannabinoid inhibitory receptors (CB1Rs), have been linked to MDD (Shen et al., 2019). It is well known that the ECS plays a key role in mood regulation, emotions, and stress response; it protects from the excitotoxic neuronal damage that impacts neuropsychiatric disorders by modulating the balance between excitatory and inhibitory activity (Rossi et al., 2009a; Ashton and Moore, 2011; Cristino et al., 2020). CB1Rs are distributed in various areas anatomically involved in mood control, particularly in the striatum, PFC and amygdala (McLaughlin et al., 2013). In a mouse model of MDD, a reduced expression of CB1Rs has been reported in the medium spiny neurons (MSN) of the nucleus accumbens, a group of GABAergic neurons expressing type-2 dopamine (D2) receptors and involved in mood-control circuitry. CB1Rs downregulation causes an increased activity of glutamatergic transmission at the level of the BLA projecting to nucleus accumbens, a key neuronal circuitry involved in MDD pathophysiology (Shen et al., 2019).

Altogether these findings suggest a complex dysregulation of neurotransmission in MDD (Figures 1, 3) and clarify the advantages of a pharmacological approach based on glutamate modulation in MDD, leading to a better knowledge of the complex neurobiology of depressive disorders and to the development of new therapeutic strategies (Gerhard et al., 2016; Lener et al., 2017).

## Inflammation-Dependent Synaptic Alterations in MDD

A growing body of evidence is now looking at the link between neuroinflammation and neurotransmitter perturbations in MDD.

As previously mentioned, cytokines released from activated microglia in the HPA-axis can modulate glucocorticoid receptor signaling and induce alteration of hippocampal synaptic plasticity in MDD and chronic stress (Pace et al., 2007; Pace and Miller, 2009; Wang et al., 2017).

The effects of proinflammatory cytokines (IL-1β and TNF-α) released by activated microglia on synaptic plasticity include alteration of NMDA and AMPA receptor subunit expression and a decrease of AMPA receptor phosphorylation (Liu et al., 2015; Riazi et al., 2015) (Figure 3). Human studies reported changes in cortical excitability as well as reduced motor evoked potential amplitudes observed following LTP- and LTD – induction protocols in MDD patients (Cantone et al., 2017). Interestingly, modifications of synaptic transmission and plasticity can in turn influence peripheral cytokine levels as suggested by clinical studies based on transcranial magnetic stimulation (TMS), a non-invasive approach able to modulate brain activity and cortical excitability (Chung et al., 2015; Croarkin and MacMaster, 2019). In a group of MDD patients, modulation of cortical GABAergic and glutamatergic imbalance by TMS protocols caused a decrease of IL-1β and TNF-α serum levels (Zhao et al., 2019), suggesting a bidirectional interaction between the inflammatory and the neurotransmitter systems.

Moreover, a recent study investigated the biological mechanism underlying the associations between the proinflammatory cytokine IL-18 and depressive symptoms related to MDD. IL-18 lacking mice showed degenerated presynaptic terminals in the molecular and polymorphic layers of hippocampal DG in association to loss of motivation at motor behavioral tests and altered neurogenesis and apoptosis (Yamanishi et al., 2019). Overall, both animal and human studies indicate a possible involvement of cytokine-mediated synaptic plasticity alterations in the cognitive deficits in patients with depression (Innes et al., 2019).

Cytokines are also able to decrease serotonin synthesis by activating the indoleamine 2,3-dioxygenase enzyme (IDO) that metabolizes tryptophan to kynurenine (Raison et al., 2010). It is well known that kynurenine itself has immunosuppressive properties, such as induction of regulatory T cells and deactivation of Th1 and Th17 cells (Lanz et al., 2017). On the other hand, high levels of kynurenine can cross the blood brain barrier (BBB) generating kynurenic acid or quinolinic acid, both active at glutamatergic synapses. Inflammatory mediators acting on microglia increase the quinolinic acid to kynurenic acid ratio, leading to net NMDA receptor agonism. While kynurenic acid blocks NMDA receptors, thus exerting a neuroprotective effect against excitotoxicity, quinolinic acid, that is increased in MDD, acts as an agonist of the NMDA receptor and directly causes glutamate release contributing to neurotoxicity (Dantzer, 2016) (Figure 3). Moreover, inflammatory mediators adversely affect astroglial expression of EAAT thus impairing glutamate removal form the synaptic cleft (Haroon et al., 2017). Finally, activated microglia products (i.e., reactive oxygen and nitrogen radicals) induce an irreversible oxidation of enzymatic cofactors for the monoamines biosynthesis, such as tetrahydrobiopterin (BH4), leading to reduction of monoamine synthesis (Kalkman and Feuerbach, 2016).

Although further investigations are needed to fully elucidate the challenging relationship between inflammation and synaptic dysfunction in depressive symptoms, we propose that an inflammatory-mediated synaptopathy might be an integral part of the pathophysiology of MDD as suggested by studies conducted in MS and in its experimental model EAE (Figure 3).

## Pathophysiology of Ms and of the Associated Depressive Symptoms

MS is an autoimmune disorder of the CNS characterized by loss of myelin sheaths and gray matter damage, followed by neurodegeneration. Although its etiology is still unclear, it is now known that interactions between susceptible genes and environmental factors are involved in disease pathogenesis. MS is mainly driven by an inflammatory cascade in the CNS, triggered by autoreactive immune cells that attack myelin and neuronal epitopes leading to demyelination and axonal degeneration. Blood–brain barrier damage allows the infiltration of immune cells, including activated T and B cells, into the CNS. The local activation of microglia and astroglia cells exacerbates the immune response and causes further damage to the neuronal cell network resulting in the genesis of demyelinating plaques and excitotoxic damage (Dendrou et al., 2015; Mandolesi et al., 2015; Reich et al., 2018) (Figures 2, 3). The manifestations of inflammatory lesions/plaques in the CNS present heterogeneous temporal and pathological patterns, resulting in a variety of neurological symptoms, including motor, sensorial, cognitive and mood disorders. It is possible to distinguish various MS phenotypes: a relapsing-remitting (RR) form, mainly characterized by clinical relapses followed by a complete recovery from symptoms in 80% of patients, and progressive forms, classified as primary-progressive (PP) starting with a partial recovery from relapses followed by an increasing rate of disability, and secondary-progressive (SP) in which the progression phase is preceded by a relapsing-remitting (RR) phase. Other important variants of disease are represented by Clinically Isolated Syndrome (CIS), in which a single clinical episode typical of MS is accompanied by a radiologically active single lesion, symptomatic or not, and Radiologically Isolated Syndrome (RIS), characterized by one or more radiologically active demyelinating lesions in absence of clinical manifestations (Vaughn et al., 2019).

### Depressive Symptoms in MS

Depression is the main psychiatric feature reported in MS patients (Di Legge et al., 2003; Marrie et al., 2015) and includes feelings of helplessness, reduced social participation and enjoyment of activities (Feinstein et al., 2014; Solaro et al., 2018). A clinical study conducted on a small cohort of RRMS patients with mood disorders reported a 38% prevalence of MDD (DSM-IV) (Byatt et al., 2011).

The main consequence of depressive symptoms is scarce adherence to pharmacological treatments or rehabilitative programs associated to worsening of functional outcomes (Binzer et al., 2019). Although these symptoms represent one of the main determinants for quality of life in MS, they are currently undervalued and undertreated in clinical practice. Depressive manifestations are part of a more complex disease and may influence other neurological and systemic symptoms, including motor, sensitive and cognitive impairment, fatigue, and pain. Furthermore, it is worth to mention that a bidirectional and detrimental interaction occurs between disease course and depression (Rossi et al., 2017) as suggested by the evidence that treatment of depression is effective in fatigue improvement (Solaro et al., 2018). Whether depressive syndrome in MS is “organic” in nature (an epiphenomenon due to increased inflammatory activity) or “reactive” (a functional reaction to neurological and physical symptoms) has been highly debated, highlighting the difficulties of its diagnosis (Feinstein et al., 2014; Rossi et al., 2017). This issue arises from the high variability of clinical measures and assessment methods used to identify clinical features of mood disorders in MS patients (Marrie et al., 2018). Therefore, it has been reported a wide range of general prevalence of depression from 4.27 to 59.6%, and of incidence from 4.0 to 34.7% in MS patients. These variable ranges are also attributable to an analysis conducted on a non-uniform population, including patients affected by RR and progressive forms of MS with an unclear distinction between active and non-active phase of the disease (Moore et al., 2012).

Of note, the risk to develop depression is two or five-times higher for MS patients than general population, with a lifetime prevalence of 50%, a rate of 44,5% during relapses but with no differences in terms of gender (Feinstein, 2011). In a recent meta-analysis, depressive symptoms have been reported in 35% of CIS and in the early phase of MS (Rintala et al., 2019). They may occur even in the absence of physical disability, sometimes anticipating the onset of clinical deficits (Haussleiter et al., 2009; Lo Fermo et al., 2010), suggesting that mood disturbances are not a reactive phenomenon to the pathology, but a considerable part of it (Feinstein et al., 2014). Furthermore, the occurrence of depressive symptoms has been associated with clinical relapses and progressive neurological disability, linking mood disturbance to immune attack and chronic neuroinflammation (McCabe, 2005; Moore et al., 2012).

Along with neuroinflammation, a combination of several predisposing factors, including environmental factors and aspects of individual personality (e.g., emotional focused, low self-esteem, maladaptive coping strategies, and other mood disturbances) are involved in the etiology of depressive symptoms in MS patients as reported for MDD (Pravatà et al., 2017; Di Filippo et al., 2018). On the contrary, some prominent risk factor such as age, sex, and family history of depression are less consistently associated to depressive symptoms in MS patients when compared to MDD (Patten et al., 2017). Differently from MDD, further studies are required to better clarify the involvement of genetic predisposing factors to depression in MS, especially concerning cytokine gene polymorphisms (Feinstein et al., 2014).

Regarding neuroimaging analysis, several MRI studies highlighted specific modifications, such as reduced cortical thickness, in the brain of depressed MS patients when compared to non-depressed MS patients. In overlap with MDD, the most involved areas include temporal pole, PFC, hippocampus with anterior and posterior cingulate cortex (Feinstein et al., 2014). Moreover, as it occurs in MRI studies conducted on MDD patients, altered connectivity between limbic system areas (i.e., amygdala and dorsolateral PFC) is extensively described in depressed MS patients (Feinstein et al., 2014; Nigro et al., 2015). On the other hand, it is worthy to note that there are radical differences between MDD and MS in their radiological features (Morris et al., 2018), considering the autoimmune etiology of MS disease. Cortical thickness and functional connectivity of the whole cortex are indeed diffusely altered in MS patients and are associated with subpial white matter demyelination and alteration of dendritic spines, the specific association between these radiological features and depressive symptoms in MS needs further investigations (Lassmann and Bradl, 2017).

The limited attention toward the mood aspects of MS symptomatology is in part due to an inadequate knowledge of their pathological basis. In this respect, some critical issues have been in part addressed by studies in animal models of MS that, despite several limitations, can reproduce some of the MS clinical and histopathological features (Ransohoff, 2012). One of the most accredited MS model is the myelin oligodendrocyte glycoprotein p35–55 (MOG35–55)-chronic EAE induced in C57BL/6 mice (Furlan et al., 2009). Such model presents different phases of the disease: the pre-symptomatic phase with absence of motor deficits, the acute phase starting from the onset to the peak of clinical symptoms and the following chronic phase, in which motor symptoms become milder. Several studies have reported depression-like behaviors in all EAE clinical phases in correlation with a number of cellular and molecular players (for review see Gentile et al., 2015). Sickness behavior (Pollak et al., 2002; Pollak, 2003) impairment in social relationships and a reduced self-preservation, expressed by social defeat and anhedonia, have been also detected in the EAE model (Rodrigues et al., 2011). EAE emotional changes have been linked to alterations of several brain regions that include the HPA (Pollak, 2003; Acharjee et al., 2013), the hippocampus (Musgrave et al., 2011; Peruga et al., 2011) and the striatum (Haji et al., 2012; Gentile et al., 2014, 2016) in association to inflammatory mediators affecting catecholamine and glutamatergic neurotransmission (Gentile et al., 2015).

## The Monoamine Theory in Depression Associated With MS

As in MDD, monoamines are important determinants in neuroinflammation-based diseases. Low tryptophan serum concentration is observed in infectious, autoimmune, and malignant diseases and disorders that involve immune cells activation due to an increase in tryptophan metabolism (Schröcksnadel et al., 2006). Accordingly, recent studies strengthen a role of serotonin in the genesis of mood disorders in MS, as demonstrated by the successful recovery from depressive symptoms with administration of SSRI in MS depressed patients (Carta et al., 2018). However, among the several pathophysiological theories at the basis of MDD, the monoaminergic hypothesis seems to impact less the course of MS. In this context, neuroinflammatory and glutamatergic theories may play a major role (Figure 2).

## The Neuroinflammatory Theory in MS Depression

The immune-mediated hypothesis of depression in MS patients has been supported by evidence from both preclinical (Chen et al., 2016; Gentile et al., 2016) and clinical studies (Gold and Irwin, 2006; Rossi et al., 2017; Lee and Giuliani, 2019).

### Cytokine Hypothesis in MS Depression

The cytokine profile of depressed MS patients is characterized by increased levels of proinflammatory cytokines, including TNF-α, IL-1β and IL-6 in both CSF and peripheral blood (Imitola et al., 2005; Rossi et al., 2017) (Figures 2, 3). Particular attention has been payed to TNF-α and IFN-γ as inflammatory mediators that drive severity of depressive symptoms during a clinical relapse in MS (Rossi et al., 2018). Similarly, peripheral levels of IL-1β in RRMS patients correlate with a negative psychiatric evaluation (Rossi et al., 2017). Furthermore, increased levels of IL-6 and decreased levels of IL-4 have been reported in depressed MS patients when compared to non-depressed MS patients and to healthy controls (Kallaur et al., 2016). This is also supported by the evidence that amelioration of depressive symptoms occurs when the inflammatory activity is attenuated (Rossi et al., 2015). Differently from MDD, CSF levels of IL-8 seems not to correlate with depressive symptoms in MS patients exposed to violence episodes during the adulthood (Brenner et al., 2018). However, further investigations are necessary to extend the cytokine profile associated to depressive symptoms in MS patients versus non-depressed MS patients, as well as to provide a comparative analysis with MDD. Few studies investigated genetic polymorphisms associated to depression in MS. In this regard, it has been reported that a favorable TNF-B1/B2 genotype is accompanied by a low inflammatory potential, decreased levels of TNF-α and increased IL-4 and IL-10 levels (Kallaur et al., 2016).

Preclinical studies investigated the link between mood disturbances and cytokines (Il-1β and TNF-α) in EAE (Mandolesi et al., 2012; Gentile et al., 2015). Regarding EAE behavioral symptoms, it is still debated whether mood disturbances are due to chronic alterations of both immune system and CNS or to an acute response to active or passive immunization used to induce EAE, resembling the LPS induced sickness behavior (Duarte-Silva et al., 2019). Differently from sickness behavior, EAE behavioral symptoms are associated to a chronic activation of HPA axis, increased inflammatory activity together with synaptic alterations (Duarte-Silva et al., 2019). These differences may suggest that EAE behavioral symptoms, and more specifically depressive symptoms, are probably not associated with sickness behavior (Duarte-Silva et al., 2019). Similarly to depressed MS patients, depressive-like symptoms in EAE have been associated to increased TNF-α and IL-1β plasmatic levels, increased inflammatory activity mediated by CD4+ and CD8+ T-lymphocytes in both peripheral blood and CNS and inflammation-mediated hypothalamic release of CRH (Duarte-Silva et al., 2019). EAE exhibits a depressive behavioral phenotype linked also to increased levels of IL-1β and TNF-α in the brain. Indeed, EAE behavioral abnormalities were reversed by blocking TNF-α or IL-1β signaling through central delivery of etanercept or IL-1ra, respectively (Haji et al., 2012; Gentile et al., 2015). These alterations have been associated to microglial activation (Gentile et al., 2015).

### The Role of Lymphocytes in MS Depression

The main difference of the immunological profile between MDD and MS is the prominent role of CD8+ T lymphocytes in the genesis of depressive symptoms in MS, where CD4+ T lymphocytes seem to have a primary role (Solaro et al., 2018). Clinical studies reported that CD8+ T lymphocyte levels in MS depressed patients are significantly increased compared to non-depressed patients, although CD4+ T lymphocytes play an important role in triggering depressive symptoms in MS, as suggested by other authors (Morris et al., 2018). CD8+ T lymphocytes are more frequently detectable in serum of MS patients during active phases (Kaskow and Baecher-Allan, 2018), and the incidence of depressive symptoms in the course of MS is more elevated during clinical relapses, consequently related to the increase in CD8+-mediated cell immunity during disease exacerbation (Tauil et al., 2018).

### The Role of Microglia in MS Depression

It is well known that activated microglia and infiltrating peripheral monocytes in CNS are crucial neuroinflammatory determinants in the pathophysiology of both MS and EAE. Together with primed T cells, they are the main inflammatory players involved in neuronal and oligodendrocyte alterations associated with synaptic dysfunction, subpial and axonal demyelination in MS/EAE (Mandolesi et al., 2015; Dong and Yong, 2019; Mecha et al., 2020). Increased microglial activity in brain regions involved in mood and cognitive control (including PFC and hippocampus) has been detected, by means of both PET and post-mortem clinical studies, in depressed MS patients in comparison to non-depressed MS patients and healthy controls (Politis, 2012). Accordingly, preclinical studies carried out on EAE investigated the link between inflammation and depressive states, revealing a direct role of microglia in the amplification of CNS neuroinflammatory response implicated in mood control (Gentile et al., 2015). In particular, depression- and anxiety-like behavior in EAE mice have been associated to the involvement of proinflammatory cytokines, such as TNF-α and IL-1β in the presence of intense microgliosis (Haji et al., 2012; Acharjee et al., 2013; Mandolesi et al., 2013; Gentile et al., 2015). Moreover, several studies demonstrated that the inflammatory milieu produced by activated microglia includes proinflammatory cytokines, prostaglandins, C3 and C4 complement proteins, reactive nitrogen species, glutamate and quinolinic acid (Gentile et al., 2015; Morris et al., 2018; Bar and Barak, 2019), suggesting a direct impact on the synaptic compartment at both structural and functional level. Of note, the inflammatory dysregulation of mechanisms responsible for synaptic homeostasis together with a dysfunction of brain area implicated in the mood control (Figures 2, 3) may occur even before the myelin damage and axonal loss in both MS patients and EAE mice (Zorrilla et al., 2001; Gentile et al., 2015; Zrzavy et al., 2017; Acharjee et al., 2018).

## Glutamatergic Theory, Structural and Functional Synaptic Alterations in MS Depression

Glutamate plays a role of primary importance both in MS and in EAE (Macrez et al., 2016) and elevated levels have been detected in the brain and CSF of MS patients with an impact on CNS integrity. Similar to MDD, an unbalance between the glutamatergic and GABAergic transmission, namely synaptopathy, has been described in several brain regions of the EAE mice and likely in MS. A long lasting synaptic pathology likely contributes to hyperexcitability and excitotoxic damage in the inflammation-driven neurodegeneration that occurs in MS/EAE (Mandolesi et al., 2015) (Figures 2, 3).

Electrophysiological studies conducted on EAE mice demonstrated that a diffuse enhancement of the glutamatergic transmission is strictly dependent on circulating pro-inflammatory cytokines, such as TNF-α and IL-1β. Of note, these synaptic dysfunctions have been detected in the gray matter not only during the symptomatic phase of the EAE disease, during which the detection of mood disturbances is challenging (Gentile et al., 2015), but also during the preclinical phases of the disease in association to depressive-like behaviors (see next paragraph). Similar alterations likely occur in MS brain as suggested by the recent development of a MS chimeric *ex vivo* models and TMS studies. In this regard, electrophysiological recordings from brain slices of healthy mice incubated with CSF of MS patients carrying elevated levels of IL-1β, revealed an IL-1β-dependent enhancement of the glutamate-mediated excitatory postsynaptic currents – through modulation of the transient receptor potential vanilloid 1channel (TRPV1) – and also an IL-1β-induced neuronal swelling (Rossi et al., 2012, 2014). Accordingly, neurophysiological measures of glutamate transmission in MS patients by TMS stimulation protocols demonstrated a positive correlation with CSF levels of IL-1β (Rossi et al., 2012). Furthermore, in a different MS chimeric *ex vivo* model, consisting in incubation of peripheral CD3+ lymphocytes derived from active RRMS patients on healthy mouse brain slices, it has been observed a TNF-α -dependent enhancement of the glutamatergic transmission similar to that observed in the EAE model (Musella et al., 2020).

Synaptopathy in the course of MS has been also associated to cognitive impairment. In fact, almost half of MS patients experience several cognitive deficits (Calabrese et al., 2011; Di Filippo et al., 2018). A preclinical counterpart has been noticed in EAE, revealing an early alteration in neurotransmission and synaptic connection without evidence of neuronal death at the hippocampal level. The excessive release of glutamate mediated by TNF-α causes the loss of the post-synaptic excitatory terminals in the CA1 region, with impairment of LTP and behavioral symptoms of memory loss and spatial disorientation (Bellizzi et al., 2016). Cognitive impairment is not only associated to a dysregulation in glutamate levels. Lower GABA levels can result in motor disability and cognitive dysfunction in MS patients, with loss of connectivity in several brain areas involved in executive functions and verbal memory (Nantes et al., 2017; Cao et al., 2018). These effects can be explained by the release of IL-1β by autoreactive lymphocytes, that has been suggested to cause a loss of synaptic inhibitory transmission in hippocampus, striatum, and cerebellum (Musella et al., 2020).

As observed for MDD, kynurenine system has been studied in depth in MS and identified as a possible biomarker for the development of disability in MS patients (Lim et al., 2017). MS patients do not differ in absolute CSF levels of tryptophan, kynurenine, kynurenic acid, and quinolinic acid compared with non-inflammatory neurological diseases but evidence shows an increase of quinolinic acid/kynurenine ratio during the relapsing phase (Aeinehband et al., 2016; Donia et al., 2019). Moreover, variable concentrations of tryptophan metabolites seem to be related to specific MS subtypes: SPMS displays a trend for lower tryptophan and kynurenic acid, while PPMS patients display increased levels of all metabolites (Aeinehband et al., 2016). It has been demonstrated that MS patients affected by depressive symptoms have higher kynurenine and lower tryptophan CSF levels (Aeinehband et al., 2016) in accordance with the above-mentioned association between depression and kynurenine levels (Raison et al., 2010) (Figure 3).

## The Inflammation-Dependent Synaptic Alterations of MS Depression

As already mentioned, most of the synaptopathy detected in the EAE brain is dependent on a direct neuronal effect of proinflammatory cytokines circulating in EAE/MS brain. The association between these inflammation-dependent synaptic dysfunction and mood alterations have been demonstrated by studies conducted in the EAE striatum during the pre-symptomatic phase of the disease or in mild-EAE mice (Haji et al., 2012; Gentile et al., 2015). The release of TNF-α by activated microglia, was demonstrated to cause an abnormal expression and phosphorylation of glutamate AMPA receptors in the EAE striatum leading to an enhancement of glutamatergic transmission during both the asymptomatic and the acute phase of the disease course (Centonze et al., 2009) (Figure 3). Accordingly, the preventive blockade of TNF-α signaling by intracerebroventricular treatment with an anti-TNF-α antibody rescued both anxiety-like behavior and synaptopathy in the EAE model. Such observations point to TNF-α as cytokine responsible of causing mood disorders mediated by glutamatergic alterations in the animal model (Haji et al., 2012).

IL-1β is also able to alter synaptic activity in EAE striatum in association to depressive-like behavior and to changing of neuronal excitability (Hewett et al., 2012; Gentile et al., 2016; Musella et al., 2020). These effects are in accordance with a burst of circulating IL-1β in EAE brain following leukocytes extravasation (Lévesque et al., 2016). Early mood disturbances have been also associated to the loss of CB1Rs-mediated control of GABA synaptic transmission in the EAE striatum, demonstrating an involvement of IL-β on CB1Rs function (Gentile et al., 2016). IL-1β is able to alter GABAergic synaptic activity by affecting CB1Rs in presynaptic compartment, not only during development of acute illness but also in a pre-symptomatic phase of disease. Moreover, IL-1β influences indirectly dopaminergic transmission in the striatum, with a detrimental over sensitization of D1 receptors and inhibition of D2 receptors that cause impairment of GABAergic activity (Gentile et al., 2016). Interestingly, EAE depressive-like symptoms were corrected by blocking IL-1β signaling through central delivery of IL-1ra, thus indicating the involvement of IL-1β in EAE depression. This effect was associated with the concomitant rescue of DA-related depressive behavior, DA CB1Rs neurotransmission and consequently to a functional rescue of striatal CB1Rs signaling (Gentile et al., 2014, 2016).

Regarding the involvement of ECS in the genesis of mood disorders in MS, many preclinical researches underlined that the stimulation of the ECS improves depressive-like symptoms in EAE mice (Poleszak et al., 2018). Clinical studies demonstrated that pharmacological activation of CB1Rs with exogenous cannabinoids causes the regression of depressive symptoms in MS patients (Ashton and Moore, 2011), suggesting a potential application in therapeutic treatments (Stampanoni et al., 2018).

It is worth noting that, besides depression, cognitive impairments are early symptoms in MS patients as a consequence of the inflammation driven dysregulation of glutamatergic and GABAergic activity. Proinflammatory cytokines are the main cause of synaptic plasticity alterations, a direct involvement of IL-1β was explored in MS patients (Mori et al., 2014) and in EAE models (Di Filippo et al., 2013; Nisticò et al., 2013).

## Clinical Insights for Cytokine Theory

In accordance with the emerging role of proinflammatory mediators in depressive states, cytokines represent potential markers as clinical response to treatment or as prognostic indicators of MDD course. For example, it has been proposed that TNF-α and IL-6 serum levels can predict a minor response to SSRI (O’Brien et al., 2007; Eller et al., 2008). Several studies conducted on SSRI and TCAs, have demonstrated a facilitation of noradrenergic neurotransmission associated with α2-adrenergic receptor activation that decreases TNF-α levels released from activated microglia in the CNS (Nickola et al., 2001; Reynolds et al., 2004). Moreover, TCAs are associated to a reduction of proinflammatory cytokines, such as TNF-α, IL-1β and IFN-γ, together with an increase of anti-inflammatory cytokines, such as IL-10, in serum of MDD patients (Maes, 1999). Interestingly, in MDD patients, that do not respond to typical antidepressant medications, elevated levels of inflammatory biomarkers were detected (Eller et al., 2008; Cattaneo et al., 2013). More specifically, lower IL-6 levels positively correlate with clinical response to antidepressant drugs (Cattaneo et al., 2013), while higher level of TNF-α are predictive of lower response to SSRI (Liu et al., 2019). The evidence suggesting a pathogenic role for proinflammatory cytokines in MDD has attracted attention on the possible use of specific anti-cytokines drugs. The antidepressant effects of drugs targeting TNF-α have already been demonstrated in preclinical models and in depressed patients suffering from autoimmune diseases. Accordingly, a recent metanalysis has shown that anti-cytokine drugs (adalimumab and tocilizumab) are associated with an antidepressant effect and increase the response to antidepressants (Kettenmann et al., 2013). Similar treatments with anti-inflammatory agents support such effect.

Interestingly, in rodent models of depression, TCA and SSRI are able to reduce the immune activation and to decrease the levels of TNF-α (Connor et al., 2000; Grippo et al., 2005). It is noteworthy that higher serotonin levels have been associated to immunomodulating effects, including T-cell and natural killer (NK) cell activation, delayed-type hypersensitivity response, production of chemotactic factors, and natural immunity derived from macrophages and microglia (León-Ponte et al., 2007; Lim et al., 2009; Dhami et al., 2013; Talmon et al., 2018).

Furthermore, the delayed effectiveness of current antidepressant drug treatment is associated with enhanced brain plasticity especially in regions commonly affected in MDD, such as the hippocampus, BLA and the PFC (Varea et al., 2012; Carceller et al., 2018). This effect could be explained by the so-called “neuroplastic hypothesis.” The activation of intracellular second messengers and the consequent effect on dendritic spine pruning may influence synaptic plasticity (Levy et al., 2018). Moreover, it has been observed that SSRI, such as fluoxetine, can reorganize the inhibitory circuitry which regulates interneural connectivity and enhance BDNF levels favoring synaptic plasticity in the hippocampus (Karpova et al., 2011). Accordingly, the role of somatostatinergic interneurons is fundamental to facilitate synaptic plasticity (Carceller et al., 2018).

Regarding treatment of MS depression, immunomodulatory and immunosuppressive drugs, namely disease modifying therapies (DMTs), such as oral dimethyl fumarate, teriflunomide, fingolimod, and intravenous drugs, such as natalizumab, and alemtuzumab (Kirzinger et al., 2013; Hunter et al., 2016; Montanari et al., 2016; Gasim et al., 2018) seem to modulate depressive symptoms in MS. Furthermore, some of these DMT drugs exert a direct beneficial effect on glutamatergic and GABAergic synaptic transmission by modulating inflammatory mediators (Gentile et al., 2016, 2018; Mandolesi et al., 2017). Although this evidence does not guarantee a unique interpretation of the relationship between DMTs and mood disorder amelioration in MS, such treatments could influence the disease course in its entirety. Regarding drugs that directly block cytokines signaling, worsening of MS symptoms was observed following treatment with anti-TNF-α due to still unclear dichotomous action of TNF-α. Drugs that act on the IL-1β system, that are effective/safe in other autoimmune diseases and show a therapeutic potential in preclinical studies have not yet been approved for MS (Musella et al., 2020).

Positive pharmacological response to SSRI, TCAs and iMAOIs in the treatment of depressive symptoms in MS may confirm that antidepressant drugs act simultaneously on both monoaminergic and glutamatergic system dysregulations. Indeed, recent studies demonstrated that SSRI treatment is effective not only on mood comorbidities related to MS, but also as a potential DMT to treat clinical relapses and progression of disability (Foley et al., 2014). Moreover, preclinical studies highlighted that administration of antidepressants, such as venlafaxine and fluoxetine, attenuates the release of proinflammatory cytokines in EAE mice (Bhat et al., 2017). These data are also supported by radiological evidence from depressed patients with MS (Grech et al., 2019). It has been demonstrated that treatment of MS patients with fluoxetine over a 24-week period is able to reduce the number of new, gadolinium-enhancing, demyelinating lesions detected at the MRI (Mostert et al., 2008). The effect of infusion drugs on depression is still a matter of debate and IFN-β or glatiramer acetate have not been associated to significant amelioration of the Beck Depression Inventory (BDI) score over 48 months of therapy (Kirzinger et al., 2013). On the other hand, the efficacy of IFN-1β on depressive symptoms has been demonstrated in another study (Comi et al., 2017). However, antidepressant treatment in combination with DMTs, particularly IFN-β, is not routinely used in clinical practice (Mirsky et al., 2016).

Finally, non-pharmacological therapeutic approaches may also improve mood symptoms in MS patients. Recent works suggest a beneficial role of physical exercise in patients with progressive MS and depression (Donia et al., 2019). In particular, it has been observed that rehabilitation is protective against neurodegeneration, improving long-term outcome and reducing the risk of depression relapses in MS patients (Joisten et al., 2019). Such effects have been related to a reduction of serum and liquor levels of proinflammatory cytokines, with a consequent modulation of glutamatergic neurotransmission and enhancement of tryptophan metabolites (Joisten et al., 2019). In EAE model, voluntary exercise improves motor disability and rescues the sensitivity of CB1Rs signaling at GABAergic synaptic terminals in the EAE striatum, suggesting a potential recovery of inflammatory mediated mood disturbances (Rossi et al., 2009b; Gentile et al., 2019).

Altogether, these observations concerning the treatment of depressive symptoms in both MDD and MS show a complex interaction between the immune and neuronal pathways suggesting the needed of further investigations, both at preclinical and clinical levels, to ameliorate the clinical outcomes.

## Conclusion

In conclusion, based on the several evidences reported here, we point to the relevance of inflammation-dependent neurotransmitter alterations as potential contributors to depressive symptoms in both MDD and MS disease. At a first glance, these complex brain disorders that diverge in multiple aspects (simply by considering MS as an inflammatory neurodegenerative disease and MDD as a neuropsychiatric disorder) seems to share few pathological aspects such as damage of brain areas devoted to mood control and some neuroinflammatory mediators. However, clinical and preclinical investigations performed in both diseases revel common traits, from the monoamine- to the glutamate- and inflammatory theories, which are often interconnected and unbalanced in the diseases (Figures 1, 2). While in MDD the contribution of inflammatory synaptic mechanisms has been mainly related to hyperactivity of the HPA axis as well as to alterations of the monoamine pathways, in MS depression, an early inflammatory synaptopathy is likely responsible of a glutamatergic and GABAergic unbalance that in the long term can cause excitotoxic damage. These observations strengthen the idea that further investigations in both pathologies are necessary to reciprocally reinforce the hypothesis that inflammation-dependent synaptic perturbations are suitable substrate for the induction of depressive symptoms in both MS and MDD at early stages (Figure 3). Unfortunately, we are not yet able to establish what are the mechanisms that drive inflammation and/or synaptic dysfunction and more studies need to be conducted.

Finally, despite we are aware that the role of inflammation in the etiopathogenesis of depressive disorders is still debated and several questions still remain open, we would like to emphasize that inflammatory cytokines are potent modulators of the synaptic compartment and that even subtle changes in their levels can induce, likely in susceptible individuals, synaptic perturbations responsible of early depressive symptoms. The synaptic glutamatergic perturbation, in different entities and with different outcomes, could represent the keystone that connects the two pathologies, joined in different ways by neuroinflammation. It is remarkable that predisposing factors, especially genetic and epigenetic factors, may play a central role to identify their common role in both diseases.

## Author Contributions

AB, ED, DC, and GM conceived and designed the manuscript. All other authors made significant contributions and approved the manuscript.

## Conflict of Interest

The authors declare that the research was conducted in the absence of any commercial or financial relationships that could be construed as a potential conflict of interest.
