# Corrigendum: Inflammation-Associated Synaptic Alterations as Shared Threads in Depression and Multiple Sclerosis

**DOI:** 10.3389/fncel.2020.647259

**Published:** 2021-01-26

**Authors:** Antonio Bruno, Ettore Dolcetti, Francesca Romana Rizzo, Diego Fresegna, Alessandra Musella, Antonietta Gentile, Francesca De Vito, Silvia Caioli, Livia Guadalupi, Silvia Bullitta, Valentina Vanni, Sara Balletta, Krizia Sanna, Fabio Buttari, Mario Stampanoni Bassi, Diego Centonze, Georgia Mandolesi

**Affiliations:** ^1^Synaptic Immunopathology Lab, Department of Systems Medicine, Tor Vergata University of Rome, Rome, Italy; ^2^Synaptic Immunopathology Lab, IRCCS San Raffaele Pisana, Rome, Italy; ^3^Department of Human Sciences and Quality of Life Promotion, University of Rome San Raffaele, Rome, Italy; ^4^Unit of Neurology, Mediterranean Neurological Institute IRCCS Neuromed, Pozzilli, Italy

**Keywords:** multiple sclerosis, major depressive disorder, excitotoxicity, antidepressant drugs, cytokines, synaptopathy, neuroinflammation, monoamine

In the published article, there was an error regarding the affiliation for authors Antonietta Gentile and Diego Fresegna. Both authors are only affiliated to affiliation 2: Synaptic Immunopathology Lab, IRCCS San Raffaele Pisana, Rome, Italy.

The authors apologize for this error and state that this does not change the scientific conclusions of the article in any way. The original article has been updated.

